# The Thermo-Optic Effect in Norbixin: Characterization by Z‑Scan and Self-Phase Modulation under CW Laser

**DOI:** 10.1021/acsomega.5c13066

**Published:** 2026-04-27

**Authors:** Nefe Jefferson Brito Silva, Thayane Portela Oliveira, Maykol Christian Damasceno Oliveira, Francisco Chagas Melo Brito, Jhaemely Gabrielly Vieira Silva, Adriano Almeida Silva, Janildo Lopes Magalhães, Hans Anderson Garcia Mejia, Francisco Eroni Paz Santos

**Affiliations:** † Physics Department and Materials Science & Engineering Graduate Program, UFPI-Federal University of Piauí, Teresina, Piauí 64049-550, Brazil; ‡ Physics Department, UFPI-Federal University of Piauí, Teresina, Piauí 64049-550, Brazil; § Materials Science & Engineering Graduate Program, UFPI-Federal University of Piauí, Teresina, Piauí 64049-550, Brazil; ∥ Supramolecular Self-Assembly Laboratory, Chemistry Department, UFPI-Federal University of Piauí, Teresina, Piauí 64049-550, Brazil

## Abstract

This work investigates the thermal nonlinear optical properties of norbixin, a natural dye extracted from *Bixa orellana* L. seeds, dissolved in acetone. The molecular structure and optical response were characterized using UV–vis, FT-IR, and Raman spectroscopies. Nonlinear optical properties were investigated using the Z-scan technique and the analysis of far-field diffraction ring patterns under continuous-wave (CW) laser illumination. The optical band gap was estimated to be 2.40 eV using the Tauc plot method, indicating that the excitation at 532 nm (*h*ν ≈ 2.33 eV) occurs in a near-resonant regime. Open-aperture Z-scan measurements revealed saturable absorption (SA) behavior, characterized by a negative nonlinear absorption coefficient of (β = −1.27 × 10^–4^ cm^2^/W), whereas closed-aperture Z-scan showed a clear peak–valley trace indicative of a strong self-defocusing effect, from which a negative nonlinear refractive index (*n*_2,th_) on the order of −5.28 × 10^–9^ cm^2^/W was determined. A discrepancy of approximately 3 orders of magnitude was observed between the nonlinear parameters obtained from Z-scan and spatial self-phase modulation (SSPM). This difference is discussed in terms of concentration-dependent thermal loading and the presence of large on-axis phase shifts. The transition from a radially symmetric to a vertically compressed diffraction pattern confirms the influence of a convection-dominated regime under high-intensity CW. These results establish norbixin as a promising sustainable nonlinear optical material for photonic applications, including optical limiters and all-optical switches, while providing fundamental insights into thermo-optic effects and convection in nonlinear media.

## Graphical abstract

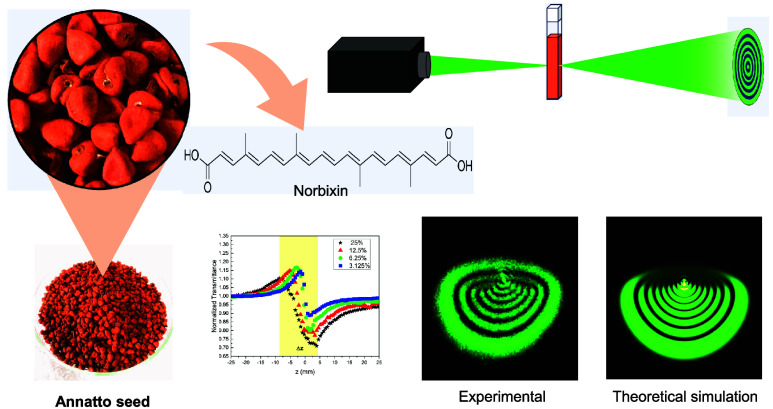

## 1 Introduction

The interplay between intense light and matter produces nonlinear optical (NLO) phenomena, enabling significant advancements in photonics, optoelectronic, and quantum technologies,^1^ including all-optical signal processing^2^ and quantum computing.^3^ Since the advent of laser technology in 1960,^4^ these phenomena, driven by thermal, electronic, and orientational mechanisms, have been extensively investigated.^5^ Thermal mechanisms, typically probed using continuous-wave (CW) lasers, involve energy absorption that induces local temperature gradients and associated changes in the refractive index­(Δ*n*).^6–9^ Recent studies have highlighted the importance of coupled thermal–optical effects and numerical modeling approaches for accurately describing laser-induced heating and thermo-optical modulation.^10^ Experimental techniques such as Z-scan, thermal lensing, and spatial self-phase modulation (SSPM) are essential for characterizing these thermally driven NLO properties.^11^ The Z-scan technique, pioneered by Sheik-Bahae et al.,^12^ quantifies the real and imaginary components of the third-order nonlinear susceptibility using a single Gaussian beam. Another approach for characterizing nonlinear optical properties is SSPM. When an intense Gaussian beam propagates through a medium exhibiting an intensity-dependent refractive index, spatially varying phase shifts are induced across the beam profile, resulting from in the formation of concentric diffraction rings in the far field.^7^ While traditional studies focus on conductive heat transfer, theoretical and experimental developments have emphasized the critical role of gravity-induced convection in determining the far-field diffraction symmetry in liquid media.^8,13–15^ Materials exhibiting strong NLO responses are critical for developing optical modulators, sensors, amplifiers, and all-optical switches.^16,17^ Organic molecules, particularly those containing extended π-conjugated systems, have emerged as promising candidates due to their high nonlinearity and structural versatility.^17–21^

In these systems, efficient π–π* electronic transitions facilitate exceptional NLO responses.^22,23^ Within this context, natural dyes such as chlorophyll-a,^24^ β-carotenoid,^25^ and various carotenoids^23,26^ serve as sustainable, biocompatible, and renewable alternatives to synthetic compounds while maintaining competitive optical performance.^27^ Norbixin, a natural yellow-red carotenoid extracted from the seeds of *Bixa orellana* L., is widely utilized in food, pharmaceutical, and cosmetic industries.^28^ Previous studies on femtosecond Z-scan technique at 1040 nm revealed competing third- and fifth-order nonlinearities in norbixin.^29^ However, the thermal nonlinear optical response of norbixin under continuous-wave (CW) excitation remains underexplored. Here, we report an investigation of the thermal nonlinearities of norbixin dissolved in acetone at 532 nm. By combining Z-scan and SSPM techniques, the study aims to (i) comprehensively characterize the nonlinear refractive index (*n*_2,th_), nonlinear absorption coefficient (β), and thermo-optic coefficients of norbixin, (ii) quantitatively resolve the influence of natural convection on beam distortion through coupled Fresnel-Kirchhoff diffraction integrals and heat transfer modeling.^8,30^ To our knowledge, this represents the first quantitative analysis of convection-induced beam distortion in norbixin, advancing the fundamental understanding of sustainable organic dyes for low-power photonic applications including optical power limiting and all-optical switching.

## 2 Experimental Section

### 2.1 Norbixin Extraction

Norbixin was extracted from 50.1 g of *B. orellana* L. seeds, using a two-step purification process as described in the literature.^31–33^ Initially, seeds were cleaned and then subjected to Soxhlet extraction with 300 mL of *n*-hexane for 24 h to remove unwanted oils. The defatted seeds were dried and transferred to a beaker containing 300 mL of 5% KOH solution, heated at 60 °C for 1 h. This alkaline treatment saponifies the predominant bixine pigment to water-soluble potassium norbixinate. After seed separation by sieving, the norbixinate solution was acidified dropwise with concentrated HCl at room temperature. This protonation step precipitates insoluble norbixin, which was filtered, washed with distilled water to pH 6, and dried in an oven at 50 °C for 24 h to yield pure norbixin powder.

### 2.2 Z-Scan Experimental Setup

To investigate the nonlinear optical properties of norbixin in acetone, we employed the Z-scan technique. The experimental setup is shown in Figure 1. A 532 nm continuous-wave (CW) laser was used as excitation source. The beam was expanded and collimated using lenses L1 and L2, then split by a beam splitter. A small portion of the beam was directed to a reference detector (D1) for power fluctuation monitoring and signal normalization. The main beam was focused using a 75 mm focal length lens (L3), generating a minimum beam waist (ω_0_) of 64 μm and a Rayleigh range *z*_0_ = 24 mm at the focal plane (*z* = 0). The sample, contained in a quartz cuvette with a 2 mm optical path, was mounted on a precision stepper motor (ESP301 Newport), allowing controlled translation along the *z*-axis through the focal region. A signal detector (D2) collected the transmitted beam after passage through an aperture positioned in the far field. In the case of the open-aperture Z-scan technique, the iris is removed and an additional lens is positioned after the sample to fully focus the beam onto the detector, enabling measurement of intensity-dependent absorption changes. This configuration allows to investigate variations in the nonlinear absorption of the material. During the experiments, the sample was exposed to varying input intensities *I*_0_, ranging from 3.10 × 10^2^ to 7.30 × 10^2^ W/cm^2^.

**Figure 1:**
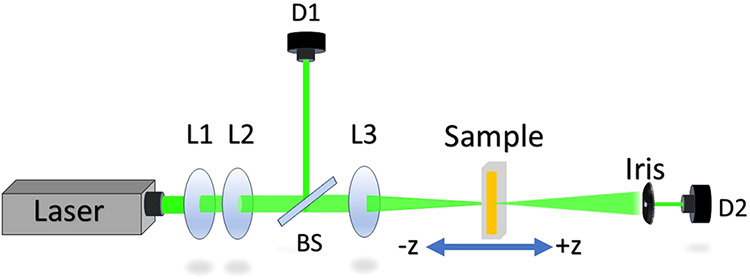
Schematic of the experimental setup for the Z-scan technique.

### 2.3 Diffraction Ring Pattern Experiments

The formation of diffraction ring patterns via SSPM is a phenomenon resulting from the spatial modulation of the refractive index induced by thermal effects in absorbing media. When a high-intensity Gaussian laser beam (I) passes through the sample, optical absorption generates a radial temperature gradient (Δ*T*) that modifies the local refractive index.^8^ For our SSPM experiment, the same 532 nm CW laser was focused by a 75 mm lens onto norbixin–acetone solutions in 2 mm-path quartz cuvettes. The front face of the cuvette was placed at the beam focus. As the beam traversed the sample, it diffracted into concentric rings due to the thermally induced phase profile. Diffraction patterns were recorded on a screen positioned 700 mm from the sample, ensuring far-field observation conditions. A schematic of this setup is shown in Figure 2. A digital camera with 30 fps resolution was used to record the ring patterns and the temporal evolution of the phase modulation patterns.

**Figure 2:**
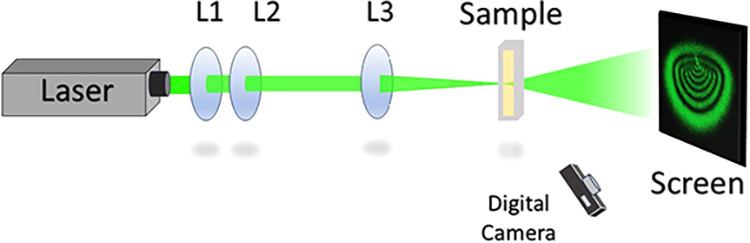
Schematic representation of the experimental setup used to generate the diffraction ring patterns.

## 3 Results and Discussion

### 3.1 Spectroscopic Characterization

The UV–Vis absorption spectrum of norbixin in acetone, obtained using a Shimadzu UV-3600 spectrometer with 1 cm optical path length, is presented in Figure 3. The spectrum exhibits three characteristic absorption peaks in the UV–Vis region (350 to 650 nm), with absorption maxima located at 435, 457, and 487 nm. These peaks correspond to π–π* electronic transitions, during which an electron is promoted from a π bonding molecular orbital to a π* antibonding molecular orbital. The energy of the light absorbed in these transitions corresponds to the energy difference between the π and π* orbitals. The highly conjugated molecular structure of norbixin is responsible for its intense light absorption in this spectral region.^28,32^ UV–Vis spectroscopy was used to determine the optical band gap (*E*_g_) of norbixin. The absorption coefficient, α, near the fundamental edge was obtained from the absorbance spectrum and analyzed with the Tauc relation for direct transitions: (*h*υ)^2^ = *A*(*h*υ – *E*_g_), where A is a constant and *h*υ is the photon energy. A linear extrapolation of (α*h*υ)^2^ versus *h*υ (Figure 3b) gives *E*_g_ = 2.40 eV.

**Figure 3:**
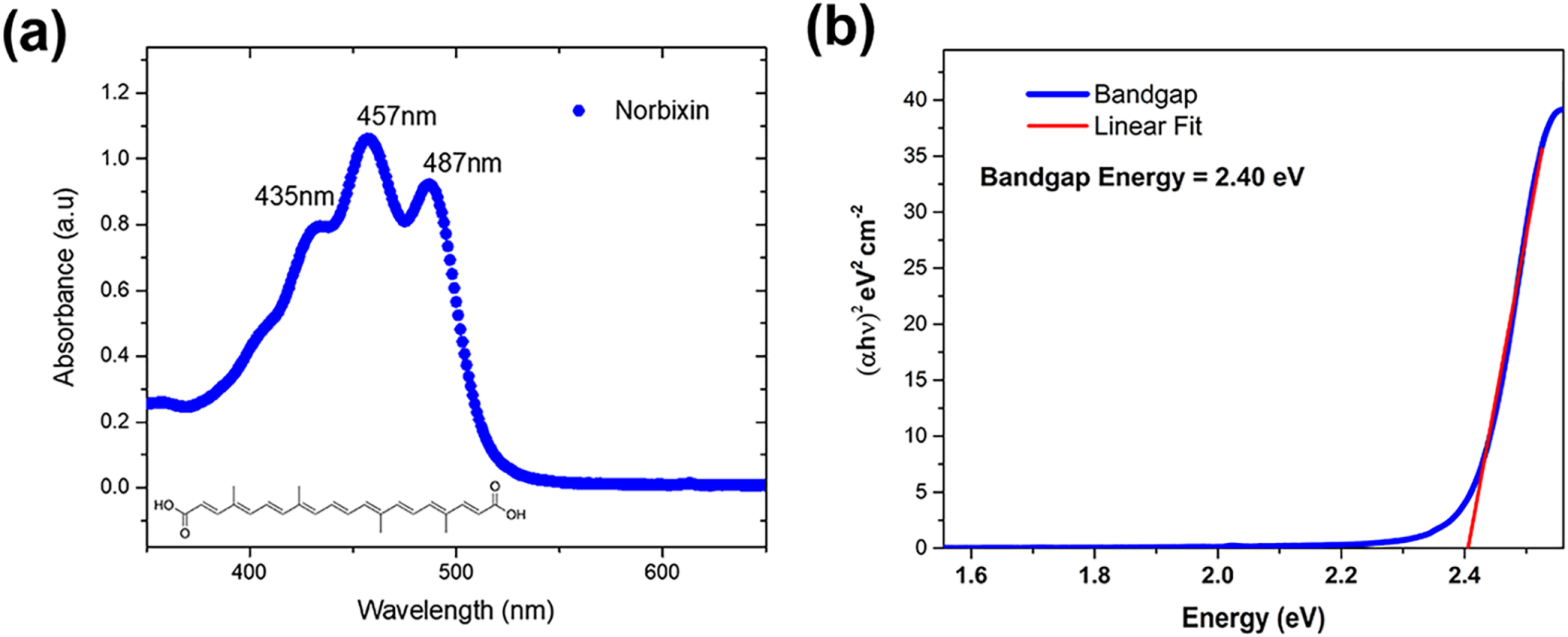
(a) UV–Vis absorption spectrum of norbixin in acetone. The inset shows the chemical structure of norbixin. (b) Tauc plot and optical band gap energy from UV–Vis data.

The infrared spectrum of norbixin is shown in Figure 4. Measurements were performed using a Bruker Vertex 70 FT-IR spectrometer in the range of 800–4000 cm^–1^, with a spectral resolution of 4 cm^–1^, averaged over 120 scans. A broad and intense band observed in the 3200–3500 cm^–1^ region is attributed to the O–H stretching vibrations of carboxylic acid groups. In the region above 2800 cm^–1^, vibrational modes associated with aliphatic C–H stretching are identified. The intense band at 1682 cm^–1^ corresponds to the C=O stretching vibrations of the carboxyl groups. Bands observed between 1610 and 1564 cm^–1^ are assigned to C=C stretching vibrations associated with the conjugated double-bond structure of norbixin.^34^ The region below 1500 cm^–1^ contains a complex set of vibrational modes that are highly characteristic of the molecular framework. Bands in the 1421 and 1274 cm^–1^ range are attributed to methyl group vibrations along the norbixin chain, while the band at 966 cm^–1^ is assigned to out-of-plane bending vibrations of trans – CH=CH– groups. The absorption peaks at 1682, 1564, 1167, and 1030 cm^–1^ are characteristic of the terminal carbonyl functionalities of norbixin.^34,35^ Overall, the FT-IR analysis provides strong evidence for the chemical transformation of the pigments. Although carotenoids occur naturally present in *B. orellana* L. seeds predominantly in the form of bixin, a lipophilic methyl ester, the absence of ester-related vibrational bands in the present samples supports their conversion to norbixin. characteristic ester bands in our samples supports the conversion of bixin to norbixin. In particular, no C–O–C stretching vibrations, typically observed in the 1255–1159 cm^–1^ region, were detected, indicating near-complete hydrolysis of the ester precursor during alkaline treatment with KOH.^36^

**Figure 4:**
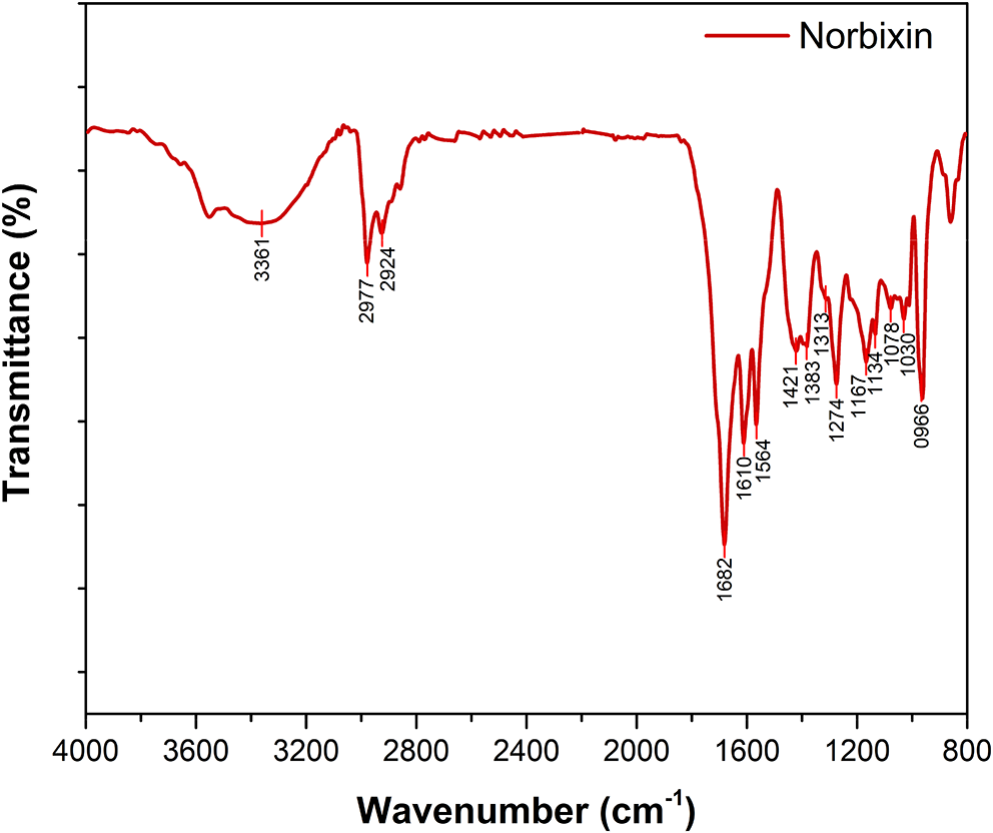
Fourier-transform infrared (FTIR) spectrum of norbixin.

Figure 5 shows the Raman spectrum of norbixin obtained in the 800–2000 cm^–1^ spectral range, using a LabRam HR-Evolution (HORIBA) Raman spectrometer with a 532 nm excitation laser. The spectra were processed using linear baseline correction followed by Savitzky-Golay smoothing. Peak positions were determined by fitting the experimental data with Lorentzian line shapes. The peak at 1010 cm^–1^ is assigned to C–CH_3_ rocking modes. The peak at 1134 cm^–1^ involves angular deformation of C=CH and C–OH groups, along with C–CH_3_ stretching and CH_3_ rocking modes. The peak at 1155 cm^–1^ corresponds to in-plane C=C–H angular deformation, whereas the peak at 1188 cm-1 is attributed to C–CH_3_ stretching from methyl groups combined with additional in-plane C=C–H angular deformation. The most intense peak, located at 1520 cm^–1^, is assigned to the C=C stretching mode of the polyene chain.^34,37^

**Figure 5:**
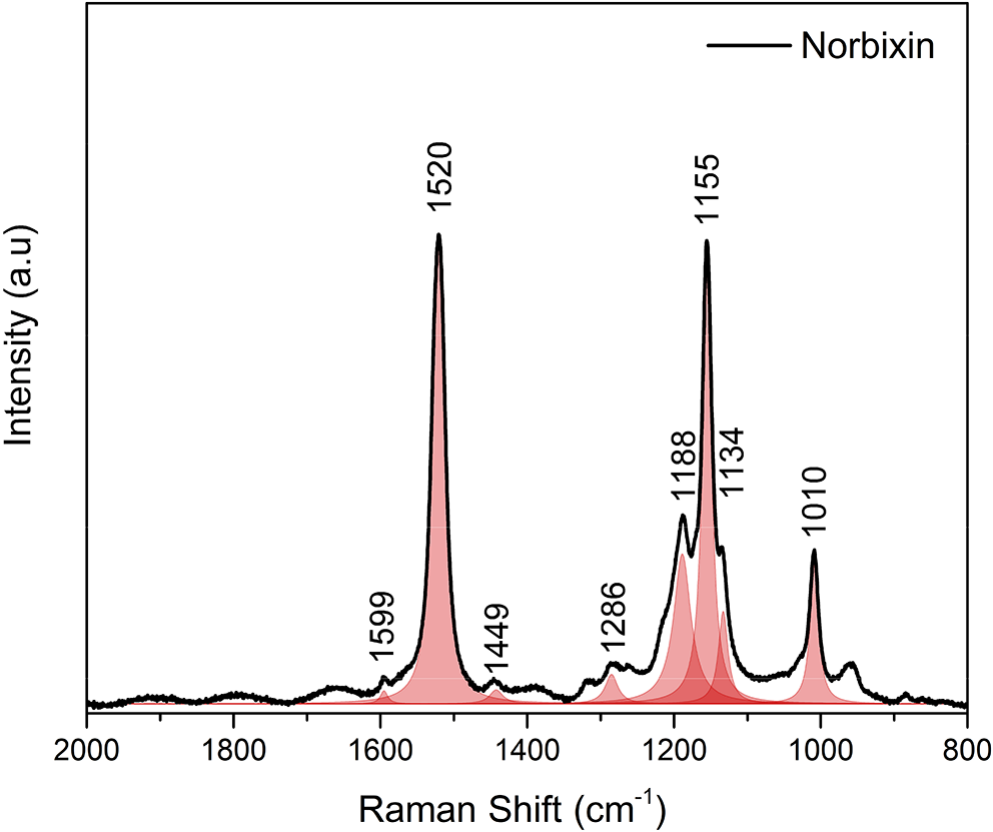
Raman spectrum of norbixin in excitation at 532 nm.

### 3.2 Z-Scan Measurements

Figure 6 shows the open-aperture Z-scan curves for a norbixin-acetone solution at various concentrations. The standard solution of 3.5 mM was defined as 100%, from which other concentrations were prepared by dilution. All curves exhibit increased transmittance as the sample approaches the focal point (*z* = 0), displaying the characteristic symmetric peak signature of saturable absorption (SA). The saturable absorption observed in norbixin arises from a fundamental competition between optical excitation and relaxation processes. At low intensities, the ground state population remains essentially unperturbed, and absorption follows Beer’s law. However, at high intensities comparable to the saturation intensity *I*_sat_, the rate of photon absorption becomes comparable to the excited-state relaxation rate. According to the nonlinear absorption model, the variation in laser intensity as it propagates through the material can be expressed as^12^

1 $$\frac{dI}{dz}=-\alpha \left(I\right)I$$

where *α­(I)* is the total absorption coefficient and *I* is the incident laser intensity at the sample position (*z*). The intensity-dependent absorption coefficient, α*(I)*, is expressed in terms of the nonlinear absorption coefficient (β) via the relationship α­(*I*) = α_0_ + β*I*. From the open-aperture z-scan data, the origin of nonlinear absorption was investigated using the following equation^12,38^

2 $$T=\frac{\ln\left(1+\frac{q_{0}}{1+x^{2}}\right)}{\frac{q_{0}}{1+x^{2}}}$$

Where *x* = *z*/*z*_0_, and $z_{0}=\frac{\pi \omega^{2}}{\lambda }$ is the Rayleigh parameter, with λ being the laser wavelength used (532 nm). Additionally, *q*_0_ = β_eff_ *I*_0_ *L*_eff_, where *L*_eff_ = (1 – exp­(− α*L*)) /α is the effective length. Here, α represents the linear absorption coefficient, and *L* is the path length of the cell containing the sample (*L* = 2 mm). The solid red line represents the theoretical fit to the data. In the open-aperture Z-scan experiment, all measurements were performed at an intensity of *I*_0_ = 7.30 × 10^2^ W/cm^2^.The calculated saturable absorption coefficient is β = −1.27 × 10^–4^ cm/W. This value is of the same order of magnitude as that calculated for a monohydrated organic salt.^38^ A qualitatively similar SA signature was observed by Kimiagar et al.^39^ in ZnO/HfO_2_ nanowires under CW excitation at 532 nm. The effect arises when the ground-state absorption cross-section exceeds that of the excited state; intense pumping depletes the ground state and increases transmission relative to the linear regime.^38,40,41^ With an optical band gap of 2.40 eV, the material is excited only 0.07 eV below resonance (λ = 532 nm; *h*ν = 2.33 eV). This quasi-resonant condition efficiently depletes the ground state, giving rise to the observed saturable absorption.

**Figure 6:**
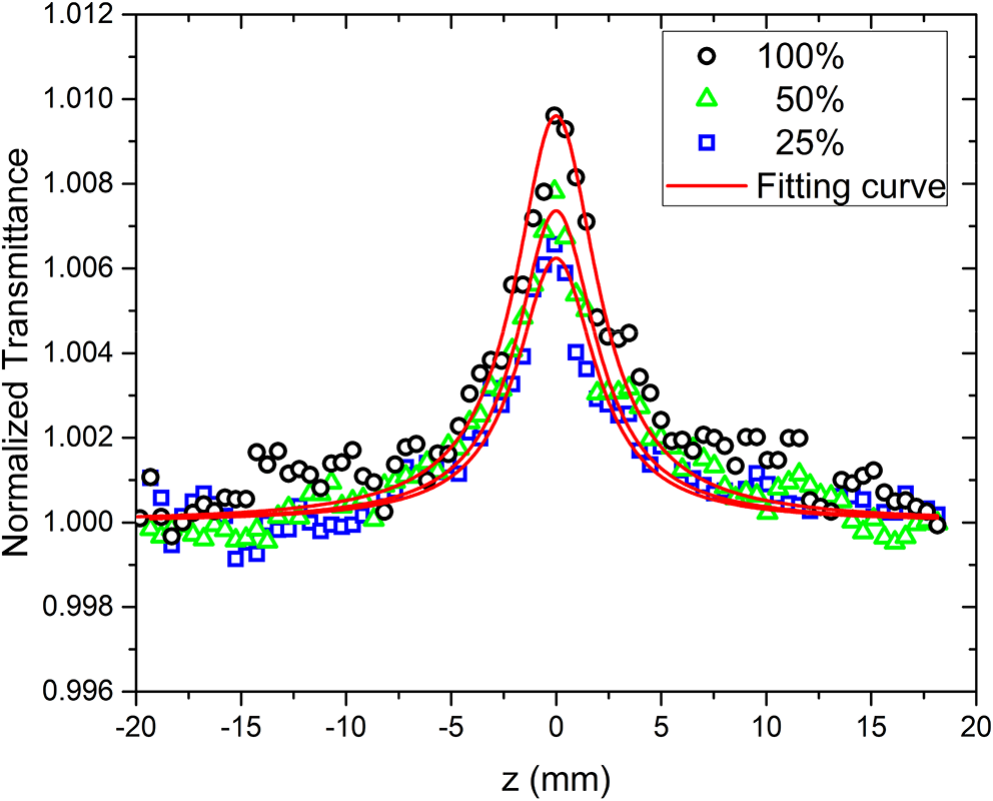
Open-aperture Z-scan curves of norbixin at concentrations of (□)­25% (0.87 mM), (Δ)­50% (1.75 mM), and (○)­100% (3.5 mM). The solid red line represents the theoretical fit by eq 2.

Figure 7 displays the closed-aperture Z-scan curves for norbixin in acetone at various concentrations. The curves present a characteristic peak–valley profile, which is attributed to a negative nonlinear refractive index (*n*_2,th_) of thermal origin, as a consequence of using a CW laser. It was also observed that the peak-to-valley separation (Δ*z*) and the asymmetry of the transmittance increase with increasing concentration. It can be observed that (Δ*z*) is 3.01 mm at a concentration of (3.125%(0.1 mM)) and increases to 12.04 mm at (25%(0.875 mM)). The pronounced asymmetry in the Z-scan curve, observed in our results, is a characteristic phenomenon of a large nonlinear phase shift regime.^26^ The fundamental cause, as demonstrated by Chen et al.,^41^ is the severe distortion imposed on the beam’s wavefront, which results in a complex redistribution of its intensity in the far field.^42,43^

**Figure 7:**
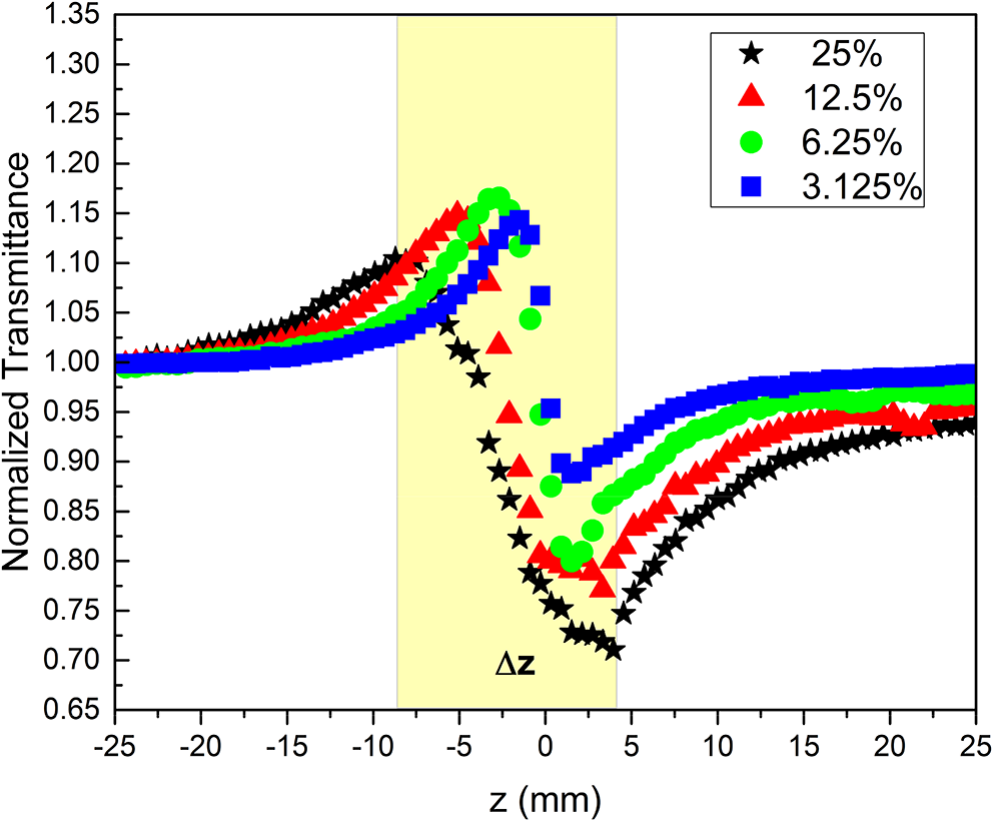
Closed-aperture Z-scan curves varying concentration of norbixin: (★) Star 25%(0.87 mM), (▲) Triangle (12.5%(0.44 mM)), (●) Circle (6.25%(0.22 mM)) and (■) Square (3.125%(0.1 mM)).

To determine the nonlinear refractive index of norbixin in acetone, closed-aperture Z-scan measurements were performed at different excitation intensities, providing access to the local, intensity-dependent nonlinear response of the medium. Figure 8(a–e) Show the closed-aperture Z-scan curves obtained for a concentration of (3.125% (0.1 mM)) under varying laser powers. The curves exhibit well-defined symmetric peak–valley profiles, and the peak–valley separation Δ*z*_p–v_ is accurately described by the theoretical model proposed by Sheik-Bahae et al.^12^ It is important to note that these measurements were conducted at relatively low concentrations, ensuring phase shifts within the validity range of the model (Δϕ<π). Consequently, the nonlinear refractive index was determined by fitting the normalized transmittance curves using the following equation

3 $$T\left(z\right)=1+\frac{4x\Delta \phi_{0}}{(x^{2}+9)\left(x^{2}+1\right)}$$

Where, Δϕ_0_ = *kn*_2_ *I*_0_ *L*_eff_ is the on-axis phase shift due to nonlinear refraction. The solid lines represent the theoretical fits, from which the nonlinear refractive index *n*_2,th_ was calculated. For a maximum intensity of *I*_0_ = 7.30 × 10^2^ W/cm^2^ the value obtained for the nonlinear refractive index was *n*_2,th_ = −5.28 × 10^–9^ cm^2^/W. The nonlinear refraction coefficient (*n*_2,th_) presents the same order of magnitude observed in the three curcuminoids^26^ and is up to 1 order of magnitude larger than that of monohydrated organic salt, also analyzed using Z-scan technique.^38^ The dependence of *n*_2,th_ as a function of the incident intensity, shown in Figure 8(f).

**Figure 8:**
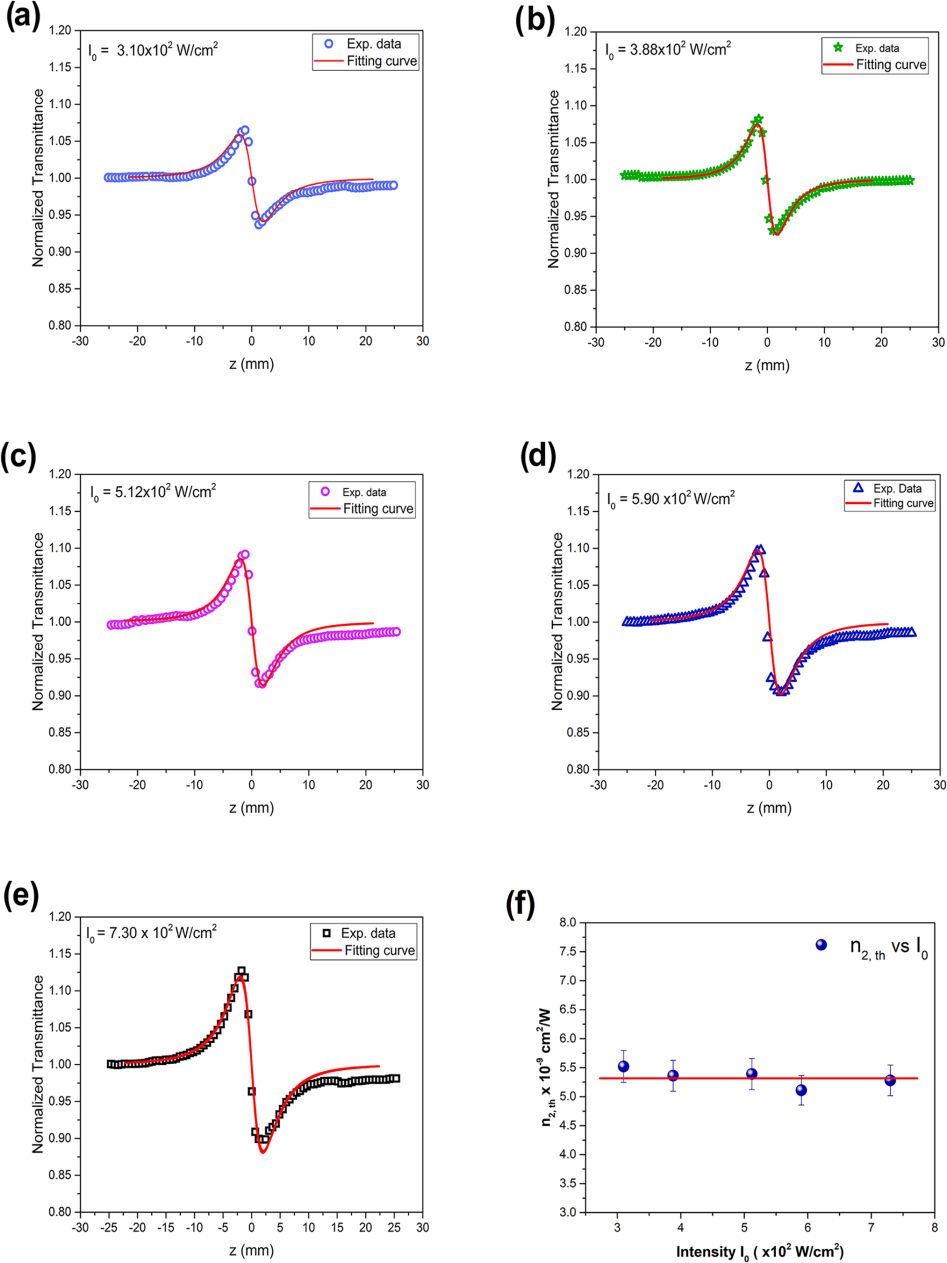
(a–e) Closed-aperture Z-scan curves of norbixin at λ = 532 nm for incident powers ranging from. The solid lines are theoretical fits. Panel (f). Calculated *n*_2,th_ as a function of incident intensity.

### 3.3 Spatial Self-Phase Modulation (SSPM) and Theoretical Model

The SSPM experiments reveal temporal dynamics that provide valuable insights into the thermal transport mechanisms governing the nonlinear optical response of norbixin. The temporal evolution of the far-field diffraction pattern associated with Spatial Self-Phase Modulation (SSPM) reflects the thermo-optic nature of the material. Figure 9 shows the evolution of the diffraction ring patterns under CW illumination, excitation at 532 nm with an incident power of 42 mW. The dynamics of the phenomenon can be divided into distinct temporal regimes. Initially, the pattern consists of a series of concentric, radially symmetric rings that progressively expand, reaching a maximum diameter within approximately 120 ms. The formation of these rings originates from thermally induced nonlinear refraction resulting from partial absorption of the incident laser energy by the sample. Owing to the Gaussian intensity profile of the laser beam, a radial thermal gradient is established, which induces a spatially varying refractive index change, Δ*n*(*r*), that acts as a divergent thermal lens. Consequently, the transmitted light undergoes **a** nonuniform phase modulation, Δφ­(*r*), leading to self-interference and the formation of the observed diffraction rings in the far-field.^8,30^

**Figure 9:**
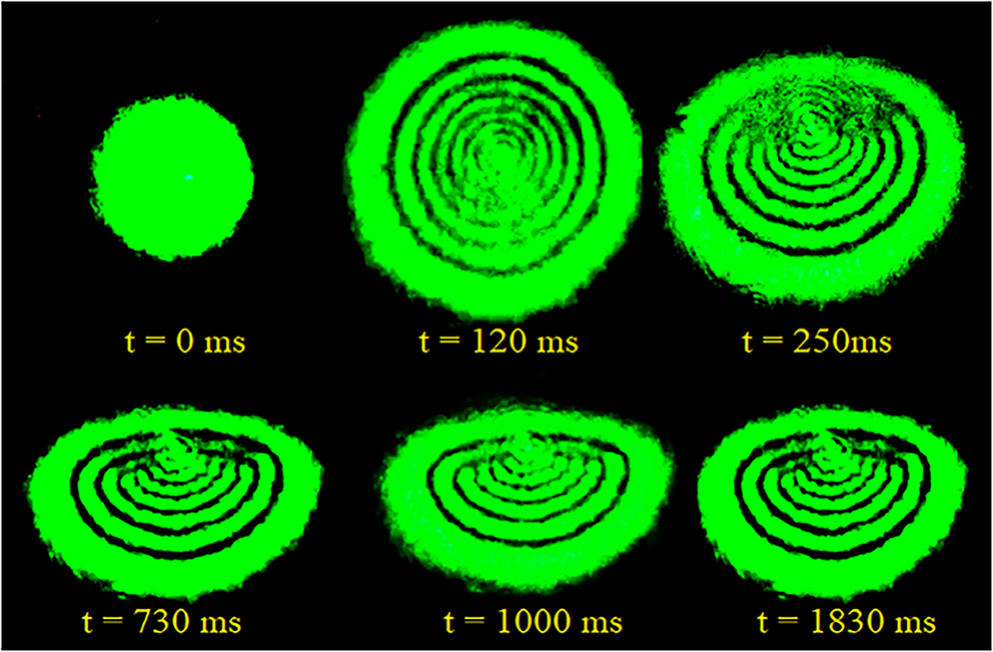
Temporal evolution of diffraction rings for norbixin solution with constant input power of 42 mW.

However, for times greater than 120 ms, a vertical symmetry breaking occurs in the pattern. The upper half of the rings undergoes a deformation, contracting toward the center of the pattern, while the lower half remains approximately constant. This observed asymmetry suggests that the system’s dynamics become dominated by thermal convection on longer time scales.^8,18,28^ In this process, buoyancy-driven convection currents transport heat vertically upward, smoothing the temperature gradient in the upper portion of the beam. Consequently, the phase modulation in this region is reduced, resulting in less light deflection, which manifests as a compression of the rings in the upper vertical direction.^30^

To quantify this dynamic behavior, Figure 10 plots the outermost ring diameter in the vertical and horizontal directions as a function of time for an incident power of 42 mW. The process can be divided into three regimes: an initial expansion phase (conduction-dominated), a deformation phase where the vertical diameter decreases while the horizontal one stabilizes (convection-dominated), and a final steady-state regime. It is observed that the horizontal diameter increases with time until it reaches a maximum value. In contrast, the vertical diameter also grows initially but, after a certain time, begins to decrease, eventually becoming smaller than the horizontal diameter.

**Figure 10:**
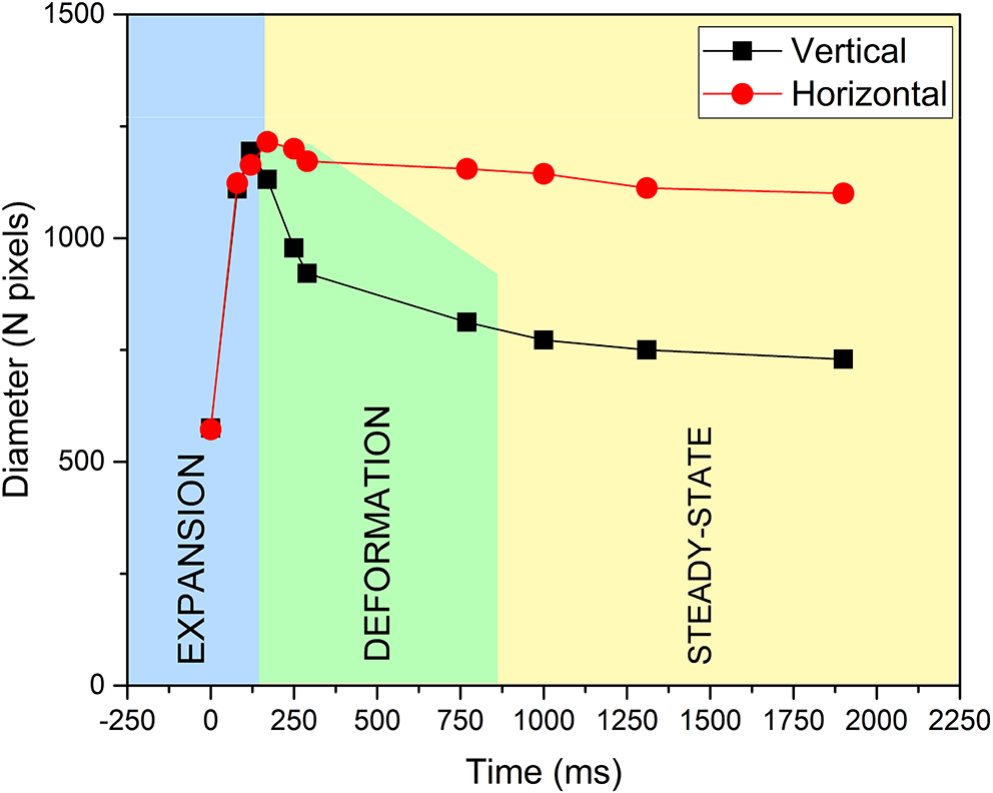
Diameter of the outermost diffraction ring in the horizontal and vertical directions as a function of exposure time for an incident power of 42 mW. The plot illustrates the expansion, deformation, and steady-state regimes.

Figure 11 shows the typical change in the diffraction ring patterns under different incident laser powers on the sample in the steady-state regime, where the rings assume a compressed and stable form. The input laser power was gradually increased from 3 mW to 47 mW. As the laser power increased, a linear increase in the number of diffraction rings was observed, as shown in Figure 12.

**Figure 11:**
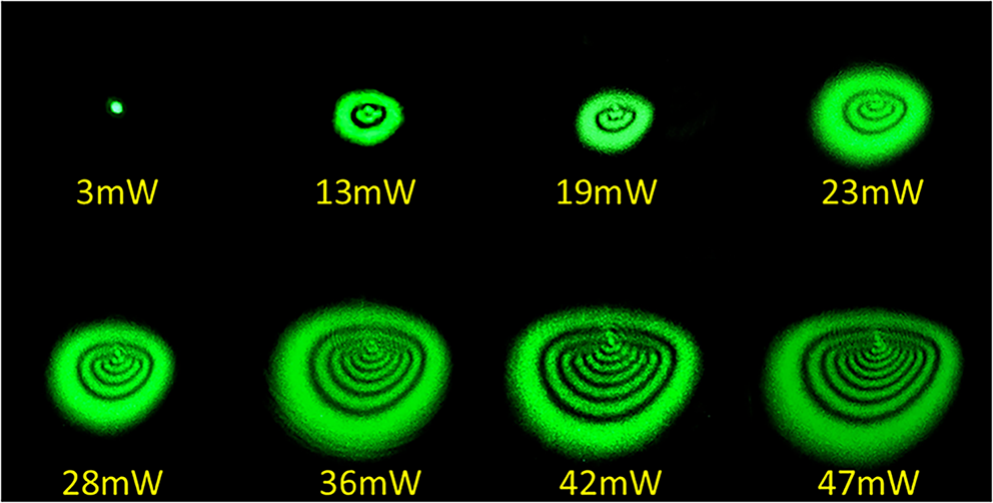
Steady-state diffraction rings of norbixin dissolved in acetone at λ = 532 nm for different laser powers at a 100% concentration.

**Figure 12:**
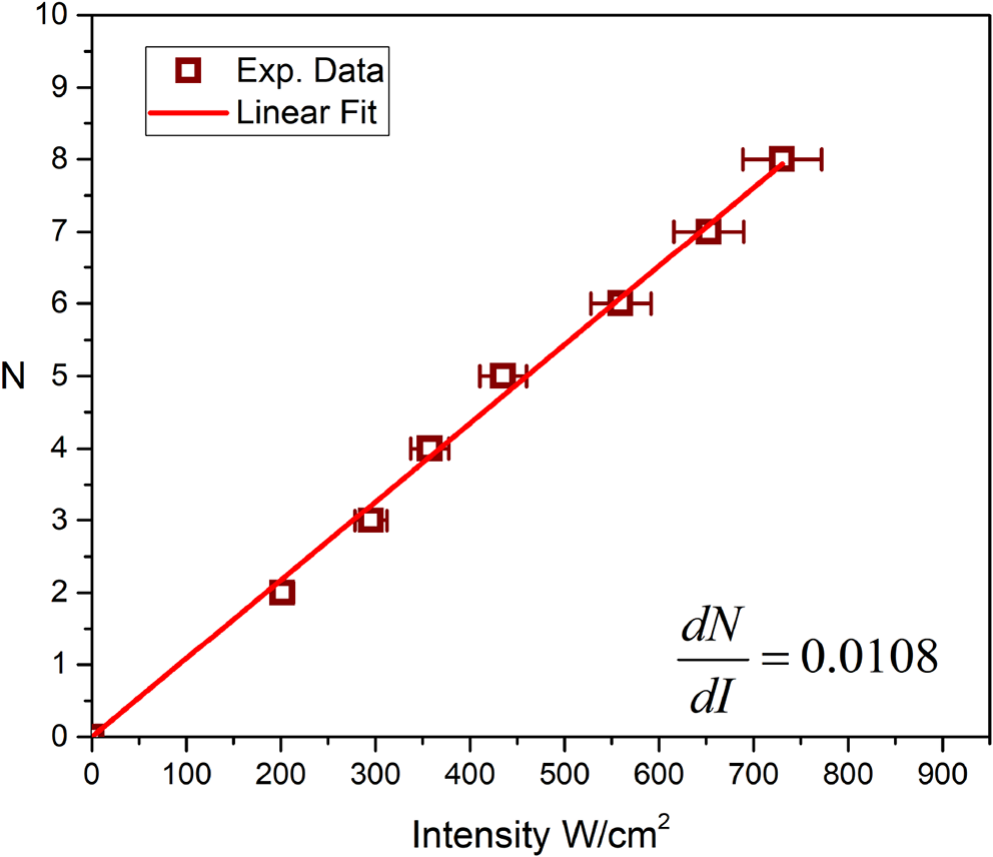
Variation in the number of diffraction rings as a function of the incident laser intensity. The square symbols are the experimental data, with a 5% error bar due to intensity fluctuations. The solid line is a linear fit to the data.

This evolution can be fully described by a theoretical model that first considers pure thermal conduction and then incorporates the effects of natural convection.

### 3.4 Regime I: Conduction-Dominated Thermal Blooming (Initial Phase, *t* < 120 ms)

Immediately after the CW laser illuminates the sample, the absorbed energy creates a localized temperature increase. In the initial moments, before bulk fluid motion can occur, heat propagates radially outward from the beam’s axis primarily via thermal conduction. For a Gaussian beam with intensity*I*(*r*) = *I*_0_exp (− 2*r* ^2^/ω_0_^2^), this creates a radially symmetric temperature profile,Δ*T*(*r*,*t*).^8,30,31^ This temperature distribution acts as a negative thermal lens, altering the refractive index of the medium according to

4 Δn(r,t)=(dn/dT)ΔT(r,t)

where *dn* /*dT* is the thermo-optic coefficient. As the beam propagates through an effective sample length*L*_eff_, it accumulates a nonuniform, but still radially symmetric, phase shift

5 $$\Delta \phi \left(r,t\right)=\left(2\pi /\lambda \right)L_{\mathrm{eff}}\Delta n\left(r,t\right)$$

This symmetric phase modulation is responsible for the self-interference that generates the concentric diffraction rings observed experimentally in the initial phase (Figure 9, *t* = 120 ms).

### 3.5 Regime II: Conduction and Convection (Steady-State, *t* > 120 ms)

As the localized heating continues, the liquid at the beam’s center becomes less dense than the surrounding fluid. This density difference, in the presence of gravity, creates a buoyant force that drives the heated liquid upward. This bulk fluid motion is known as natural convection. To model this, we follow the formalism developed by Karimzadeh,^30^ which modifies the standard heat transfer equation to include a convective flow term. Assuming a uniform upward convection velocity *v*_*x*_ along the vertical (*x*) axis, the heat transfer equation becomes

6 $$\rho C_{p}\left[\partial \left(\Delta T\right)/\partial t+v_{x}\partial \left(\Delta T\right)/\partial x\right]={k\nabla }^{2}\left(\Delta T\right)+Q\left(r\right)$$

Here, ρ is the density, *C*_p_ is the specific heat capacity, *k* is the thermal conductivity of the solvent, and *Q*(*r*) = α*I*(*r*)­is the heat generated per unit volume due to absorption.

The crucial element in eq 6 is the advection term, *v*_*x*_∂(Δ*T*)/∂*x*, which describes the transport of heat by the bulk fluid motion. The solution to this equation, as derived by Karimzadeh^30^ using a Green’s function approach, gives the time- and space-dependent temperature profile

7 $$\Delta T\left(x,y,t\right)=\frac{\alpha P}{\pi \rho c_{p}}\left\{\int_{0}^{t}\frac{dt}{8Dt^{\prime }+\omega^{2}}\exp\left(-2\left[{\left(x-v_{x}t\right)}^{\prime 2}+y^{2}]/[8Dt^{\prime }+\omega^{2}\right]\right)\right\}$$

Where *P* is the total laser power, *D* = *k*/ρ*C*_p_ is the thermal diffusivity, and ω_0_ is the beam waist. The presence of the (*x* – *v*_*x*_ *t*’)­term in eq 7 is the mathematical origin of asymmetry. It shows that the center of the temperature distribution is displaced upward along the *x*-axis, breaking the radial symmetry. This asymmetric temperature profile Δ*T*(*x*, *y*, *t*)­leads to an asymmetric refractive index profile Δ*n*(*x*, *y*, *t*), which in turn generates the vertically compressed diffraction rings observed experimentally in the steady-state regime (Figure 9, *t* > 250 ms and Figure 11).

After the beam propagates through the nonlinear medium, the complex amplitude of the field at the output plane is described by means of the Fraunhofer approximation applied to the Fresnel-Kirchhoff diffraction integral. Thus, the intensity profile at the far-field observation plane is expressed as a function of the spatial coordinates *x*′ and *y*′, where *d* is the distance between this plane and the sample output (eq 8).^30^

8 $$I\left(x^{\prime },y^{\prime },t\right)={\left|\begin{array}{l} U_{0}\frac{i\pi \omega^{2}}{\lambda d}\exp\left(\mathrm{idk}\right)e\mathrm{xp}\left(-\frac{\alpha L}{2}\right)\times \int_{-\infty }^{\infty }dx\int_{-\infty }^{\infty }dy\exp\left(-\frac{x^{2}+y^{2}}{\omega^{2}}\right)\\ \times \exp\left[i\left(k\frac{x^{2}+y^{2}}{2R}+\Delta \phi \left(x,y,t\right)\right)\right]\times \exp\left[\mathrm{ik}\frac{xx^{\prime }+yy^{\prime }}{d}\right] \end{array}\right|}^{2}$$

### 3.6 Quantitative Analysis from Steady-State Rings

Despite the spatial asymmetry caused by convection, the magnitude of the nonlinear effect can be quantified from the total number of rings N observed in the steady-state. Each ring corresponds to a 2π phase difference between the beam center and its edge. The total on-axis phase shift, Δϕ_0_, is therefore related to the number of rings by *N* = Δϕ_0_/ 2π.^44^ Combining this with the on-axis phase shift definition, Δϕ_0_ = (2π/λ)*L*_*eff*_Δ*n*_0_, and the effective nonlinear relation we arrive at the working equation

9 $$N=\left(L_{\mathrm{eff}}/\lambda \right)n_{2,\mathrm{th}}I_{0}$$

This model, while based on an aberration-free lens approximation, provides a robust method to determine the strength of the nonlinearity from the experimentally measured linear dependence of *N* on *I*_0_ (Figure 12). By rearranging (eq 9), the effective nonlinear refractive index *n*_2,th_ is calculated from the slope of the linear fit^11,45^

10 $$n_{2,\mathrm{th}}=\frac{\lambda }{L_{\mathrm{eff}}}\frac{\mathrm{dN}}{\mathrm{dI}}$$

Furthermore, the fundamental thermo-optic coefficient *dn* /*dT* can be linked to *n*_2,th_ through the steady-state heat conduction model, which yields the relation^11^

11 $$\frac{\mathrm{dn}}{\mathrm{dT}}=\frac{4n_{2,\mathrm{th}}\kappa }{\alpha_{0}\omega^{2}}$$

Using the experimentally determined slope *dN* /*dI* = 0.0108 *cm* ^2^/*W*and our known system parameters, (eqs 10 and 11) allow for the calculation of both *n*_2,th_ and *dn* /*dT*. (see Table 1).

**Table 1:** The Calculated NL Optical Parameters of the Norbixin in Acetone

Technique used	*n*_2,th_ (cm^2^/W)	*dn*/*dT*(k^–1^)	β (cm/W)
Z-scan	–5,28 × 10^–9^	–7.68 × 10^–6^	–1.27 × 10^–4^
SSPM	–3,23 × 10^–6^	–4.70 × 10^–3^	__

Discrepancies in the *n*_2,th_ values were observed when comparing results from diffraction ring patterns with those obtained via Z-scan. Some studies attribute such variations to differences in intensity levels employed across techniques.^26,46^ While the refractive index is intensity-dependent, in our case identical intensity levels were used. The measurements were performed at different concentrations, which may also contribute to the discrepancy in the *n*_2,th_ values obtained by the two techniques. An increase in norbixin concentration significantly enhances the linear absorption coefficient, which in turn escalates the cumulative thermal load within the acetone solution. To contextualize the magnitude of the nonlinear coefficients obtained herein, Table 2 compares our values with those reported for representative natural dyes and related photonic materials under CW excitation.

**Table 2:** Compares the Values Obtained in This Work with Those Reported for Representative Natural Dyes and Related Photonic Materials under CW Excitation

Sample	Wavelength (nm)	SSPMn_2_ (cm^2^W^–1^)	Z-scan *n*_2_ (cm^2^W^‑1^)	β (cm W^–1^)	refs.
lycopene	650		–7.26 × 10^–8^	–2.0 × 10^–4^	^23^
Monohydrated organic salt	520		–3.32 × 10^–8^	–1.59 × 10^–4^	^38^
Curcumin	473	–5.80 × 10^–7^	–7.49 × 10^–9^		^26^
Dimethoxycurcumin	473	–4.10 × 10^–7^	–5.94 × 10^–9^		^26^
Chlorocurcumin	473	–2.49 × 10^–7^	–4.68 × 10^–9^		^26^
Rose oil	532	–0.30 × 10^–6^	–0.92 × 10^–8^		^46^
Linseed oil	532	–0.43 × 10^–6^	–1.35 × 10^–8^		^46^
Chamomile oil	532	–1.32 × 10^–6^	–1.98 × 10^–8^		^46^
Carmoisine dye	532		–7.25 × 10^–8^	–0.41 × 10^–4^	^47^
β-carotenoid	635		–2.00 × 10^–7^	–0.63 × 10^–4^	^48^
Norbixin	532	–3.23 × 10^–6^	–5.28 × 10^–9^	–1.27 × 10^–4^	This work

The findings from the independent Z-scan and SSPM methods provide compelling evidence that the nonlinear optical response of norbixin under CW laser illumination is thermally dominated, with its dynamics fully explained by the transition from a conduction-driven to a convection-driven regime.

Figure 13 displays simulated far-field diffraction patterns in a Python environment for a laser beam propagating through a liquid with a fixed convection velocity of 2.5 mm/s, following Karimzadeh’s model.^30^ The simulation incorporated beam parameters and sample properties including thermal conductivity, *K* = 0.179 W/mK, density, ρ = 79 kg/m^3^, specific heat, *c*_p_ = 2150 J/kgK, and absorption coefficientα = 1.13 cm^–1^. Comparison with experimental results (Figure 9) reveals discrepancies in the initial time. As previously observed by Karimzadeh,^30^ such deviations stem from approximations in the convection velocity value. The physical properties of the solvent (acetone), specifically its specific heat, thermal conductivity and density were explicitly included in the simulations. Some discrepancies in early time dynamics reflect approximations in the convection velocity model and transient thermal effects not fully captured by the steady-state approach.^49^ Additional experimental factors such as beam fluctuations at onset may further influence ring formation. Collectively, these physically justified factors account for the observed deviations between simulated and experimental patterns.

**Figure 13:**
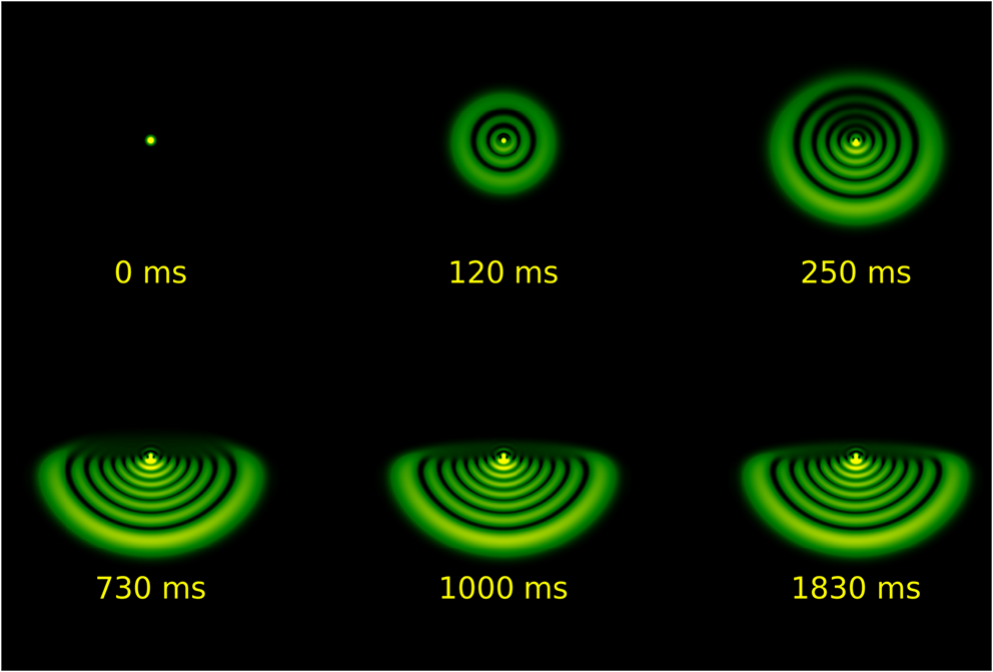
Temporal evolution of the diffraction rings was analyzed for an incident power of 42 mW and a wavelength of 532 nm, considering a convection velocity of *v*_*x*_ = 2.5 mm/s.

Figure 14 illustrates the diffraction ring patterns for input powers ranging from 3 mW to 47 mW, calculated numerically in Python, using the laser and sample parameters mentioned above, and considering a fixed time of 1 s, corresponding to the steady state.

**Figure 14:**
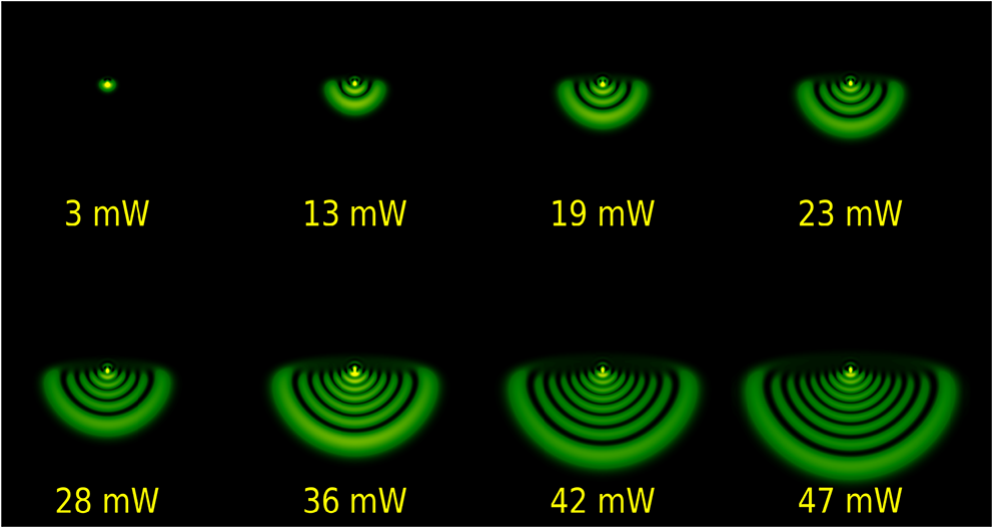
Simulation of the diffraction rings observed with different input laser powers in the far field.

The numerical results obtained (Figure 14) are in good agreement with the experimental data obtained in the steady state (Figure 11). On the other hand, as shown in Figure 13, the simulated rings are symmetric in the initial times, which is also observed in the experimental patterns. Moreover, from 730 ms onward, the simulation faithfully reproduces the observed patterns, indicating that the model based on thermal conduction and convection accurately describes the steady-state and deformation regimes of the system.

## 4 Conclusions

This study establishes norbixin, a natural dye extracted from *B. orellana* L., as a highly responsive thermo-optic nonlinear material under continuous-wave (CW) excitation at 532 nm. Open-aperture Z-scan measurements revealed saturable absorption (β < 0), arising from ground-state depletion, while closed-aperture Z-scan experiments demonstrate a pronounced self-defocusing behavior (*n*_2,th_ < 0), with magnitudes comparable to those reported for high-performance organic chromophores. The three-orders-of-magnitude discrepancy observed between the *n*_2,th_ values obtained from Z-scan and SSPM measurements is attributed to the higher concentrations employed in the SSPM configuration, which enhance linear absorption and increase the cumulative thermal load. This enhanced thermal deposition drives a transition from conduction-dominated to convection-dominated heat transport, leading to the experimentally observed symmetrical breaking and vertical compression of the far-field diffraction rings.

These experimental findings are further supported by the estimated optical band gap of 2.40 eV, confirming that excitation at 532 nm occurs in a quasi-resonant regime, thereby facilitating the large nonlinear optical response. Moreover, Fresnel–Kirchhoff diffraction modeling incorporating convective heat transfer successfully reproduces the steady-state asymmetry observed in the diffraction patterns. Collectively, these results position norbixin as a sustainable and renewable alternative to synthetic nonlinear optical materials, with strong potential for applications in optical limiting and low-power all-optical switching.
